# Influence of soil moisture regimes on growth, photosynthetic capacity, leaf biochemistry and reproductive capabilities of the invasive agronomic weed; *Lactuca serriola*

**DOI:** 10.1371/journal.pone.0218191

**Published:** 2019-06-28

**Authors:** Aakansha Chadha, Singarayer K. Florentine, Bhagirath S. Chauhan, Benjamin Long, Mithila Jayasundera

**Affiliations:** 1 Centre for Environmental Management, School of Life and Health Sciences, Federation University Australia, Mount Helen, Victoria, Australia; 2 Centre for Crop Science, Queensland Alliance for Agriculture and Food Innovation (QAAFI), The University of Queensland, Gatton, Queensland, Australia; 3 School of Science, Engineering and Health, RMIT University, Bundoora, Victoria, Australia; Instituto Agricultura Sostenible, SPAIN

## Abstract

Global temperatures are predicted to increase by 1.5–5.9°C during this century, and this change is likely to impact average rainfall, with predictions that water deficit will perhaps be the most severe threat to sustainable agriculture. In this respect, invasive weeds, which have traits better adapted to drought stress than crops, add to concerns regarding crop sustainability. *Lactuca serriola*, an aggressive agronomic weed is thought to be a successful weed because of its ability to maintain high water use efficiency under drought conditions. In this study, experiments were conducted to examine the influence of different soil moisture regimes (100%, 75%, 50% and 25% water holding capacity (WHC)) on growth, photosynthetic capacity, leaf biochemistry and reproduction of this species. Soil moisture significantly affected plant’s height, stem diameter, number of leaves and biomass. The highest plant height (115.14 cm ± 11.64), shoot diameter (9.4 mm ± 0.18), leaf area (1206.5 mm^2^ ± 73.29), plant fresh weight (83.1 ± 3.98) and dry weight (22.38 ± 1.24) were recorded at 75% soil moisture content. A fundamental adaptation to drought was observed as plants in the 25% WHC treatment had the highest root: shoot ratio. Soluble sugars and total phenolic content were highest in the 25% WHC treatment and significantly different to 100% WHC which was a response to soil moisture stress to ameliorate the damaging effects of reactive oxygen species produced under stress conditions. Results also indicate that *L*. *serriola* can survive and produce seeds under water stress as more than 6000 seeds were produced per plant in all WHC treatments. In this study, there was no significant difference in the seed weight, number of seeds produced and their germination ability. This can have a huge impact on agricultural systems as the species can survive both under low and high soil moisture conditions. We therefore suggest that the demonstrated ability of *L*. *serriola* to complete its life cycle and produce biomass and seeds under water stressed conditions leads to the introduction of strategies that minimize weed survival while maximizing irrigation efficiency for the crop. A clear understanding of the ecological and biological characteristics of this weed will help land managers take appropriate control measures to mitigate the effect of this species on economic crop productivity.

## Introduction

Plants undergo or display symptoms of extreme water deficiency when the required levels of moisture are unavailable in their habitat soil. This happens when the plants continuously lose water via transpiration or evaporation due to high temperatures and the loss of ground moisture is not refurbished [1]. This extreme dryness, declared as drought, which extends to long periods of time, is prevalent on a global scale. These conditions combine lack of water through rain with high temperatures and radiation, and currently pose the most important environmental threats to plant survival and crop productivity [2]. The prevailing stress conditions are exacerbated by competition from associated weed species, due to photosynthetic decrease, constraint of metabolic processes and interference with nutrient availability [3, 4]. With regard to the outcomes of competition for water in a cropping situation, it depends on the abilities of the crop and weed species to survive under water stress conditions [5]. It has been noted that invasive plant species, by virtue of their traits, are more adaptable to water stress than crop and pasture species [6]. Thus, prevailing arid conditions is an important factor in weed invasion, as it impacts the competitive establishment, physiology, subsequent growth and reproduction of the mixture of plants in a crop [7], making it an important element of crop production studies.

The ability to survive drought differs between species, within species and the stage of development of a plant based upon intensity and duration of water stress conditions [1, 2, 8]. It is known that moisture deficiency is characterized primarily by drought signalling in roots, reduction in leaf water potential, closure of stomata and cellular dehydration [9]. Secondary or long-term effects of soil moisture stress are reduction in cell enlargement and growth, reduction of cellular and metabolic activities, photosynthetic inhibition, turgor loss, manufacture of reactive oxygen species, and altered carbon partitioning [1, 9, 10].

It has been established that drought stress is an important limiting factor for plant growth and establishment. This prevents plants from achieving the maximum growth potential set by their genotypes [1, 5]. Growth is accomplished through cell enlargement, cell division and differentiation and involves a complex interaction of genetic, physiological, ecological and morphological events. Moisture stress affects these events as impaired mitosis and loss of turgor results in limited cell division and obstructed cell elongation respectively which in turn causes diminished growth [11, 12]. During water stress, production of abscisic acid triggers stomatal closure, following which, there is a decline in intercellular CO_2_ levels, and therefore, a photosynthesis reduction [10, 13]. Simultaneously, metabolic changes occur in photosynthetic pigments and components [14, 15], and there is a reduction in the functioning of Calvin cycle enzymes which all together results in reduced plant growth and yield [16].

Prevailing macro and micro environmental factors, such as water stress conditions, trigger survival instincts in plants, which produce changes in their biochemical processes as an adaptive measure. Under moisture stress, production of reactive oxygen species increases and as an adaptive measure, plants produce enzymatic antioxidants to limit the oxidative degradation. In order to maintain their cellular hydration, plants synthesize and accumulate solutes by osmotic adjustment, which function as osmolytes in cells and help in the preservation of cellular structure, its components and the protection of metabolic functions [17, 18].

Environmental factors acting to the detriment of plant health have an increased impact while the plant is in its growth phase and reduces the reproductive allocation to seeds, which could result in the production of either fewer or smaller seeds. They could also influence seed quality traits such as seed dormancy and chemical defence [19, 20]. Water deficiency at the time of seed maturation also impacts the dormancy and germination of weed seeds [21]. Thus the adaptive measures incorporated by a plant as a response to stressed conditions including carbon assimilation, allocation of photo assimilates to different parts and the preservation of its reproductive ability, all contribute to the endurance of a plant species under environmental stress [22].

It has been claimed that the ability of *Lactuca serriola* to adapt to a varied macro and micro environmental conditions in several countries is responsible for its successful establishment and proliferation [23, 24]. Although *L*. *serriola* has been established and is growing in several disparate climatic conditions, the interactions between *L*. *serriola’s* growth and surrounding environmental factors such as soil moisture content, soil salinity levels and weather and climatic conditions, have not been quantified until quite recently. Understanding the influence of various soil moisture regimes on *L*. *serriola* is an essential requirement to help us to predict its proliferation, consequent impact and develop suitable containment methods.

Recent climate modelling studies have shown that among significant future environmental factors, rainfall events will decline and droughts will increase in frequency in the state of Victoria (Australia) [25]. While it is known that the water content in the soil of a particular area or region largely determines and aides the establishment of any invasive weed species [5, 26]. However it is obvious that different species will respond differently when exposed to water stress conditions. Indeed, some weed species have survived and even thrived to the extent of completing their life cycle, maintaining growth and their reproductive ability even during times of severe water scarcity [26, 27]. Therefore, it is to our benefit to log, observe and study the impacts of water stress on all plants, especially invasive weeds, which in turn will lead to improved management practices and for possibly altering the fate of economically important vegetation under climate change [2]. Hence, the present study was performed to gauge and enumerate the morphological, photosynthetic physiological, biochemical and reproductive responses of *L*. *serriola* to various soil moisture regimes. The objectives of the study were to: (i) observe and evaluate the growth and reproductive abilities of *L*. *serriola* to four different (100%, 75%, 50% and 25%) soil moisture regimes; and (ii) study the underlying physiological and biochemical changes in response to moisture stress. This would help to assess if this species is directly impacted with the possibility for greater establishment and proliferation under future drought conditions.

## Materials and methods

### Experimental design

The experiment was conducted using a Completely Randomized Design located within a temperature-controlled glasshouse at Federation University Australia’s Mt Helen campus in Australia from December 2017 to April 2018. The glasshouse was maintained at 26/18°C day/night temperature and 50%-60% relative humidity.

**Fig 1:**
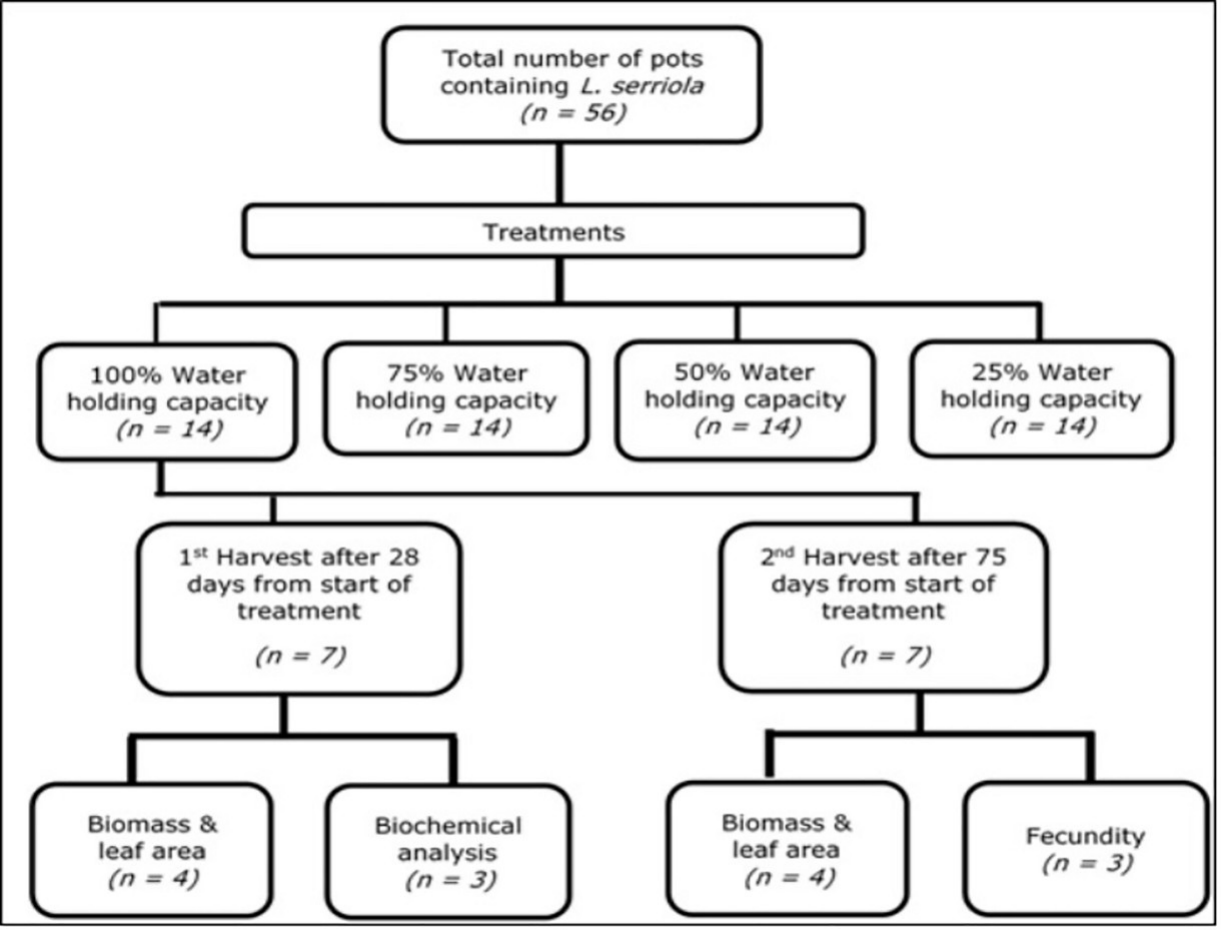
Experimental layout for the treatment and harvesting of *L*. *serriola* plants. The first and second harvest described for 100% WHC treatment was similarly followed for 75%, 50% and 25% WHC treatments.

The experiment comprised of 56 pots in total, each containing one *L*. *serriola* seedling. At the start of the treatment, the pots were randomly selected and marked for the treatments. As detailed in Fig 1, out of the 56 seedlings, 14 were allocated to each treatment, with a water holding capacity at 100%, 75%, 50% and 25%. The 14 pots in each treatment were then split into two groups of seven and marked for two harvests, the first harvest was at 28 days when the plants were in their vegetative growth stage and the second harvest was at 75 days, when the plants were in the reproductive stage. Of the seven plants harvested in the first harvest from each treatment, four were used for biomass analysis and leaf area measurement and the other three were used for biochemical analysis. Of the seven plants harvested in the second harvest, four were used for biomass analysis and leaf area measurement, and the remaining three were used to collect seeds for fecundity testing.

The net photosynthesis rate, stomatal conductance, maximum photochemical yield of Photosystem II (*F*_*m*_*/F*_*v*_ value) and chlorophyll content (SPAD readings) were also measured at the first and second harvest. The pots marked for the second harvest were also used to take repeated measurements for shoot height, shoot diameter, and leaf count, so that measurements could be recorded until the experiment concluded.

### Seed collection and seeding establishment

*Lactuca serriola* seeds were collected in April 2016 from an abandoned agricultural land in Werribee, Victoria (37^O^ 82’, 144^O^ 57’). Seeds were manually separated from approximately 100 plants, cleaned and placed in labelled air-tight glass bottles. These glass bottles were stored at room temperature in the seed ecology lab at Mt Helen campus of Federation University until used.

During December 2017, a total of 56 black plastic pots (19 cm diameter and 18 cm height) were filled with 2500 g of soil mixture (2:1 mixture of field soil and potting mix). Of the soil used, 500 g soil (roughly 5 cm of the pot’s top soil) was autoclaved. Out of the five seeds sown, the seedlings, once established, were thinned down to a single, vigorous plant at the six leaf stage.

### Water holding capacity

Water holding capacity of the soil was determined using a modified method based on Bajwa *et al*. [28]. Three kilograms of soil was placed in each of three pots (19 cm diameter and 18 cm diameter height), which were then saturated with tap water. The pots were covered with a black plastic sheet, then left to drain for 48 hours without disturbance. After 48 hours, three samples of soil weighing 300 g each, from the mid-section of each pot was taken. Each sample was then dried in an oven at 90°C for 72 hours followed by recording the dry weight of each sample. The soil sample’s water holding capacity (WHC) was the difference between weights of the wet soil and dry soil. The 75%, 50% and 25% WHC levels were determined as a fraction of the 100% WHC found.

Once the rosettes were established, the soil moisture treatment commenced where *L*. *serriola* plants were grown in four different soil moisture levels, as determined by varied soil WHC, 100% (control), 75%, 50% and 25%. To re-establish the appropriate WHC, each pot was weighed using an electronic digital balance every alternate day and appropriate quantity of water slowly added, to the soil’s surface. To measure the weight of the growing plants, additional plants (in addition to the 56 pots used in the experiment) were grown to record their weight at varying developmental stages. The treatment was conducted for 85 days, until the plants were developed fully and had produced seeds.

### Morphological measurements

To analyse growth parameters, the seven plants marked for the second harvest were repeatedly measured for shoot height, shoot diameter and leaf count. These measures were taken once per week from the start of the treatment until the 63^rd^ day. The shoot height (cm) was measured (surface of the soil to tip of the bud) with a small carpenter’s tape. The shoot diameter (mm) was measured with a digital calliper at the pot rim level and the number of leaves on each plant were counted manually.

### Physiological parameters

Gas-exchange measurements were done on the newest, fully expanded, undamaged leaf from each plant, using a LI-6400 XT photosynthesis system (LI-COR Inc., Lincoln, LE, USA), which is a portable infra-red gas exchange system, with an incorporated leaf chamber. Three plants were randomly selected for the gaseous exchange investigation. To take the measurements, the leaf chamber was set with an air flow per unit leaf area of 500 μmol s^-1^, leaf temperature of 22–23°C, ambient thermal reading of 20–23°C with humidity at 30%. Net photosynthesis and the stomatal conductance was recorded after each leaf had reached a steady state, where assimilation and stomatal conductance had stabilized, which required 2 to 4 minutes.

The maximum photochemical yield of Photosystem II (*F*_*m*_*/F*_*v*_) was measured using a MINI PAM (MINI–PAM II Photosynthesis Yield Analyser., WALZ Mess-und Regeltechnik., Germany). The measurements were taken directly from each plant on three fresh and fully expanded, dark adapted leaves. The leaves were allowed 10 minutes to become dark adapted before the measurements were taken.

The *F*_*m*_*/F*_*v*_ value was calculated using the formula:

Fm/Fv=(Fm−Fo)/Fm

Where *F*_*m*_ is the maximal fluorescence obtained with a 0.8 saturation flash and *F*_*0*_ is the dark fluorescence yield. The three *F*_*m*_*/F*_*v*_ readings were then averaged to produce a single comparative estimation of *F*_*m*_*/F*_*v*_ for each plant.

### Biochemical parameters

To measure the total soluble sugar and total phenolic content, healthy and undamaged leaves were collected from three plants in each treatment at the first harvest at 28 days. The total weight of the leaves from each plant was noted immediately. These leaves were dehydrated in a dehydrator (Sunbeam Food Lab Dehydrator, Model number DT 6000) at 35°C for 48 hours, and the dry weight recorded. These leaf samples were stored at 4°C until analysed. The experiments were conducted in triplets for the reliability of data. The total soluble sugar content of each sample was determined using the Phenol sulphuric method of DuBois *et al*. (1956) [29], as improved by Lee and Kim (2000) [30]. Total phenolic content was determined using the Folin-Ciocalteu method, as described by Javanmardi *et al*. (2003) [31].

To estimate each leaf’s chlorophyll content, a chlorophyll meter, SPAD– 502 (Soil-Plant Analyses Development, Konica Minolta Sensing, Inc, Japan: Model number 72923021) was used. Three fresh and fully developed leaves from each plant were randomly selected to take the readings, which were averaged to produce a single comparative estimation of chlorophyll content for each plant.

### Reproductive parameters

Mature seeds were collected from each plant during the experiments, kept in labelled bags at room temperature in the seed ecology lab at Federation University’s Mt Helen campus. Seed weight was determined by assessing subsamples of 100 seeds from each treatment.

The flowers on each plant were counted at 85 days from the commencement of the WHC treatment. The number of seeds produced on 10 randomly selected flowers from each plant was counted. The result was multiplied by the number of flowers on each plant to obtain an approximation of the number of seeds produced per plant.

To understand the influence of various soil moisture regimes on seed germination of the seeds produced by plants subjected to water stress, three replicates of 20 seeds was taken from pooled seed samples collected from each treatment. The seeds were surface sterilized by rinsing them in 1% sodium hypochlorite for 1 minute and then rinsed thoroughly with RO water. 20 randomly selected seeds were placed evenly into a 9 cm diameter Petri dish lined with Whatman No. 11 filter paper. Sterilized distilled water was added to each Petri dish, which was subsequently sealed with para-film to prevent loss of water. Petri dishes were placed into an incubator (Thermoline Scientific Australia, Temperature and humidity Cabinet, Model: TRISLA-495-1-SD), maintained at 30/20°C day/night temperature with a 12 hr light/12 hr dark and 24 hours darkness photoperiod, with cool-white fluorescent lamps that produced a photosynthetic photon flux of 100μmol m^-2^s^-1^. Germination was logged daily with seeds showing a radicle length of 2 mm were labelled germinated. Seed germination was conducted only in 30/20°C temperature regime, as previous temperature studies (unpublished data), had shown that optimum germination (90%) was achieved in this temperature range.

### Harvesting

During both harvests, four plants from each treatment were used to record the total fresh and dry biomass of each plant by weighing the leaves, stem and roots. To calculate root: shoot ratio, the root and shoot lengths were measured at each harvest. Leaf area was measured separately for each plant using a Planimeter (Paton Electronic Planimeter developed in conjunction with CSIRO. Serial number 711-14-531/21). The leaves, stem and roots were dehydrated in an oven at 70°C for 48 hours, to obtain a constant weight for measurement of the total dry biomass.

### Statistical analysis

Relative Growth Rate (RGR) was calculated for shoot height or shoot diameter using the formula:

RGR=(ln(W2)−ln(W1))/(t2−t1)

where *ln* is the natural logarithm and *t*2 (time 2) is *t*1 (time 1) + one week and *W*1 and *W*2 are the variables in *t*1 and *t*2, respectively.

Data were analysed using R (Team R Core 2016) [32]. Normality of data was confirmed using the histogram of residuals and no transformation was required as all the data passed through normality test. The *post-hoc* analysis was performed using the least square means function which are represented by different letters whereby different letters represent significant differences amongst the treatments at the alpha level indicated. An adjustment using Tukey’s HSD was made for multiple comparative studies. Repeated measures ANOVA was performed throughout the experiment on the data for relative growth rate of shoot height, shoot diameter and number of leaves. One-way ANOVA was performed for the rest of the data. Appropriate graphs were generated for each of the analysis using Microsoft Excel to visually represent the treatment factors and their interaction.

## Results and discussion

### Influence of soil moisture on growth

**Fig 2:**
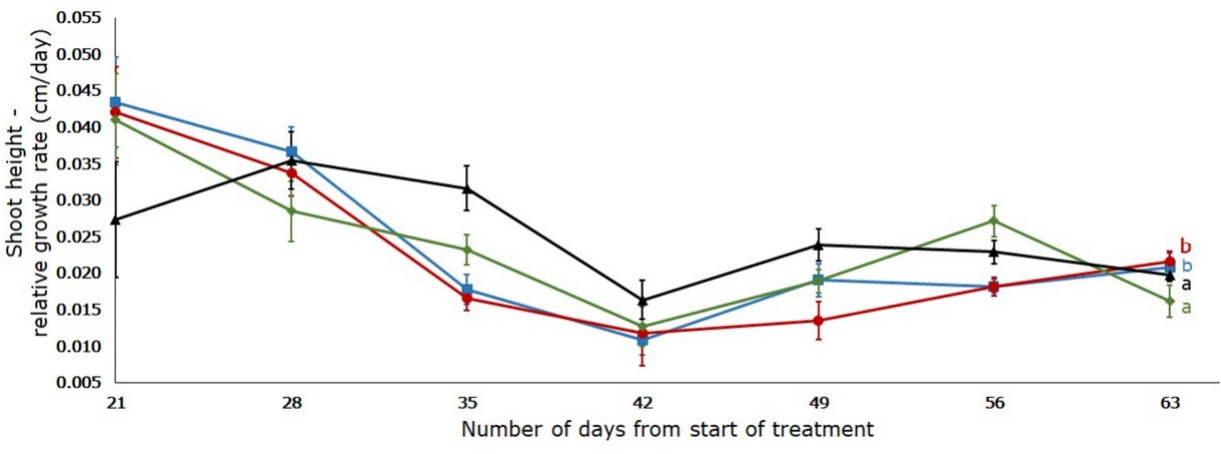
Effect of four different soil moisture levels on the relative growth rate of shoot height of *L*. *serriola*. Shoot height was measured weekly from day 14 until day 63 of the treatment. 25% (▲) WHC, 50% (◆) WHC, 75% (●) WHC and 100% (■) WHC represent 25%, 50%, 75% and 100% soil water holding capacity in which the plants were maintained. Vertical bars indicate standard error. Different letters represent significant differences amongst the treatments at p < 0.05 (n = 7) throughout the entire experiment.

Soil moisture significantly affected (p < 0.05) the plant’s height, stem diameter, number of leaves and biomass. All the plants continuously gained height from week one until the last day of the experiment (Fig 2).

On the 21^st^ day, plants had a height of 15.57cm (± 2.91), 18.74 cm (± 2.76), 10.19 cm (± 1.12) and 7.36 cm (± 1.29) in the 100%, 75%, 50% and 25% WHC, respectively. Plants in 75% WHC treatment (40.21 ± 3.88 cm) reached double the height of plants in the 25% WHC (20.33 ± 1.94 cm) treatment on the 35^th^ day. However, on the 63^rd^ day, the tallest shoot height was observed in the 75% WHC treatment (115.14 cm ± 11.64), followed by the 100% (104.71 cm ± 8.61), 50% (77.71 cm ± 7.35) and 25% (76.78 cm ± 6.64) WHC treatments, respectively. There was no significant difference in the plant’s height in the 25% and 50% WHC treatment (p < 0.05). Similarly, plant’s height in the 75% and 100% WHC treatments were not significantly different (p < 0.05) (Fig 2).

**Fig 3:**
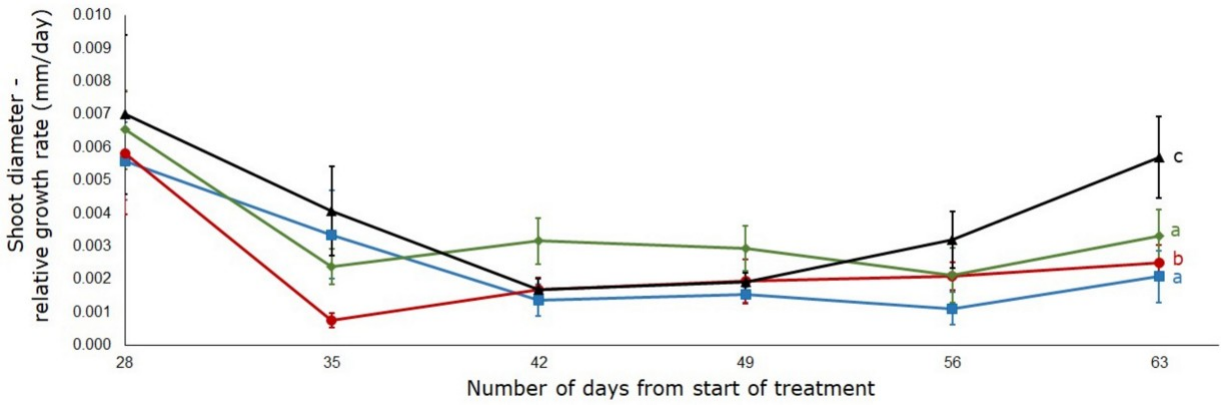
Effect of four different soil moisture levels on the relative growth rate of shoot diameter of *L*. *serriola*. Shoot diameter was measured weekly from day 28 until day 63 of the treatment. 25% (▲) WHC, 50% (◆) WHC, 75% (●) WHC and 100% (■) WHC represent 25%, 50%, 75% and 100% soil water holding capacity in which the plants were maintained. Vertical bars indicate standard error. Different letters represent significant differences amongst the treatments at p < 0.05 (n = 7) throughout the entire experiment.

The shoot diameter increment over time showed a similar trend (Fig 3). On day 28 which was the first day of observation, plants had a shoot diameter of 7.56 mm (± 0.35), 8.13 mm (± 0.10), 6.74 mm (± 1.19) and 5.84 mm (± 0.28) in the 100%, 75%, 50% and 25% WHC treatments. At day 63, the largest shoot diameter (9.4 mm ± 0.18) was observed in the 75% treatment followed by 8.75 mm (± 0.22), 8.44 mm (± 0.28) and 7.62 mm (± 0.39) in the 100%, 50% and 25% WHC treatments, respectively. Shoot diameter in the 100% and 50% WHC were significantly (p < 0.05) different to plants in the 75% and 25% WHC treatments (Fig 3).

**Fig 4:**
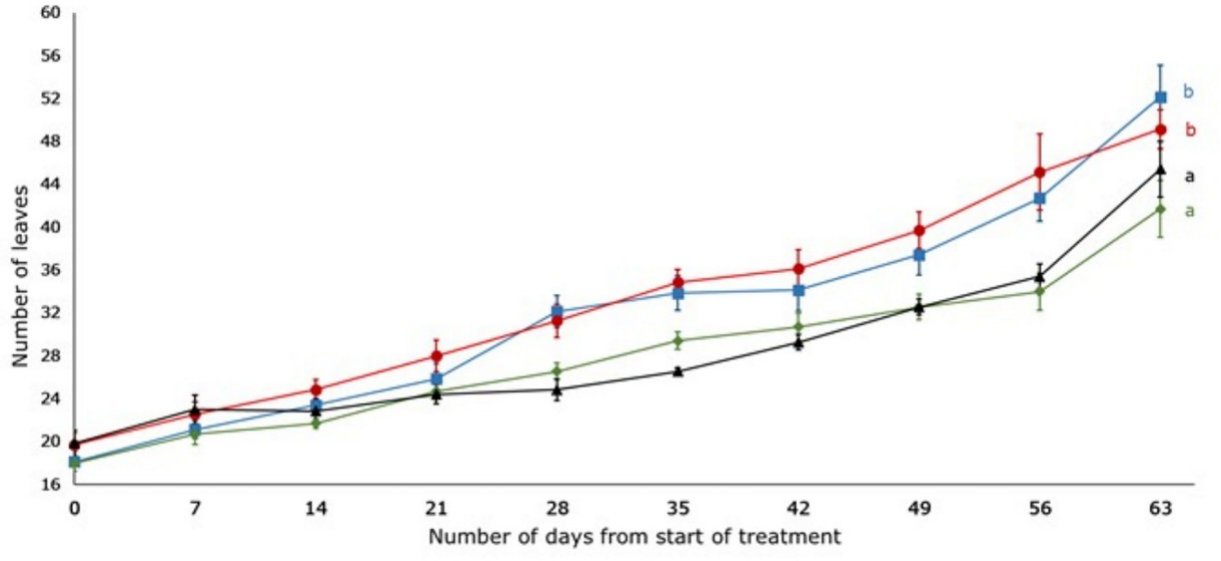
Effect of four different soil moisture levels on the number of leaves of *L*. *serriola*. Number of leaves was measured weekly from the start of the treatment until day 63. 25% (▲) WHC, 50% (◆) WHC, 75% (●) WHC and 100% (■) WHC represent 25%, 50%, 75% and 100% soil water holding capacity in which the plants were maintained. Vertical bars indicate standard error. Different letters represent significant differences amongst the treatments at p < 0.05 (n = 7) throughout the entire experiment.

The number of leaves was higher in the 100% and 75% WHC treatments and significantly different to 50% and 25% WHC treatments (p < 0.05) (Fig 4). The largest number of leaves was observed on the last day of observation (63^rd^ day) in the 100% WHC treatment (52.14 ± 2.96), followed by the 75% (49.14 ± 1.82), 25% (45.43 ± 2.61) and 50% (41.74 ± 2.66) WHC treatments, respectively (Fig 4). As evident from Fig 4, from the start of the treatments until day 35, each plant added between 1 and 3 leaves per week, but between day 35 and day 63, there was an increase of 3 or more leaves per week.

Leaf area was measured at the end of the first and second harvesting periods. In the first harvest, plants in the 100% WHC treatment had the greatest leaf area, followed by the 75%, 50% and 25% WHC treatments. However, during the second harvest, the 75% WHC treatment plants had the greatest leaf area followed by the 50%, 100% and 25% WHC treatments. Significant differences were found for the leaf area, being significantly greater for the 75% and 100% WHC treatments (Tables 1 and 2). At the first harvest, the 25% WHC treatment had the highest root: shoot ratio, followed by the 50%, 75% and 100% WHC treatments (Table 1). At the second harvest, the 25% WHC treatment still had the highest root: shoot ratio, however, it was followed by 100%, 50% and 75% WHC treatments (Table 2).

**Table 1:** Effect of four different soil moisture levels on the leaf area, root: shoot ratio, total fresh weight, total biomass, net photosynthesis, stomatal conductance, *Fm/Fv* value and SPAD value of *L*. *serriola* at first harvest.

Parameters	25% WHC	50% WHC	75% WHC	100% WHC
**Leaf area (mm**^**-2**^**)**	392.46 (57.74) ^a^	408.17 (48.60) ^a^	658.73 (109.73) ^b^	828.89 (33.69) ^b^
**Root: shoot ratio**	0.66 (0.07) ^b^	0.62 (0.13) ^ab^	0.44 (0.09) ^ab^	0.33 (0.04) ^a^
**Total fresh weight (g)**	31.68 (1.29) ^a^	47.73 (2.66) ^b^	50.99 (4.47) ^c^	57.61 (1.51) ^c^
**Total dry weight (g)**	5.22 (0.64) ^a^	5.17 (0.15) ^a^	7.91 (0.56) ^b^	8.31 (0.23) ^b^
**Net photosynthesis** **(mol m-2s-1)**	6.23 (0.65) ^a^	6.51 (0.03) ^a^	10.71 (0.45) ^b^	11.44 (1.77) ^b^
**Stomatal conductance**	-0.002 (0.002) ^a^	0.003 (0.001) ^b^	0.01 (0.001) ^c^	0.011 (0.004) ^c^
**Fm/Fv value**	0.81 (0.004) ^a^	0.81 (0.003) ^a^	0.82 (0.005) ^ab^	0.81 (0.004) ^a^
**SPAD value**	45.54 (0.61) ^a^	41.56 (0.79) ^b^	40.38 (0.49) ^b^	41.49 (0.85) ^b^

Values are mean (± standard error) at 1^st^ harvest. Different letters indicate means are statistically different when tested with Tukey’s HSD at p < 0.05.

**Table 2:** Effect of four different soil moisture levels on the leaf area, root: shoot ratio, total fresh weight, total biomass, net photosynthesis, stomatal conductance, *Fm/Fv* value and SPAD value of *L*. *serriola* at second harvest.

Parameters	25% WHC	50% WHC	75% WHC	100% WHC
**Leaf area (mm-2)**	825.07 (57.19) ^a^	954.77 (107.25) ^a^	1206.5 (73.29) ^b^	928.23 (30.41) ^b^
**Root: shoot ratio**	0.4 (0.03) ^b^	0.28 (0.02) ^ab^	0.26 (0.03) ^ab^	0.29 (0.01) ^a^
**Total fresh weight (g)**	59.18 (2.47) ^a^	59.64 (1.04) ^b^	83.1 (3.98) ^c^	70.72 (3.20) ^c^
**Total dry weight (g)**	17.15 (0.84) ^a^	17.48 (0.60) ^a^	22.38 (1.24) ^b^	19.38 (0.32) ^b^
**Net photosynthesis** **(mol m-2s-1)**	22.73 (3.71) ^ac^	19.90 (2.69) ^b^	24.68 (1.55) ^c^	25.30 (1.90) ^c^
**Stomatal conductance**	0.03 (0.02) ^a^	0.003 (0.009) ^b^	0.033 (0.02) ^a^	0.035 (0.01) ^a^
**Fm/Fv value**	0.80 (0.003) ^a^	0.81 (0.006) ^b^	0.82 (0.003) ^bc^	0.81 (0.003) ^b^
**SPAD value**	45.41 (0.90) ^a^	43.58 (0.36) ^b^	42.47 (0.64) ^bc^	43.60 (0.94) ^b^

Values are mean (± standard error) at 2^nd^ harvest. Different letters indicate means are statistically different when tested with Tukey’s HSD at p < 0.05.

In the first harvest, total fresh weight was highest in the 100% WHC treatment, followed by 75%, 50% and 25% WHC treatments (Table 1). However, in the second harvest total fresh weight of the 75% WHC treatment was the highest, followed by 100%, 50% and 25% WHC treatments (Table 2). During the first harvest, plants in the 100% WHC treatment had the highest total dry biomass, followed by the 75%, 25% and 50% WHC treatments (Table 1). However, in the second harvest, the 75% WHC treatment had the highest dry biomass, followed by the 100%, 50% and 25% WHC treatments (Table 2). The 50% and 25% WHC treatments had similar amounts of dry weight, which were significantly different to the 75% and 100% WHC treatment (p < 0.05) (Tables 1 and 2). Results obtained from this study show that shoot length, number of leaves, total leaf area, fresh weight and dry weight of *L*. *serriola*, all saw a drop when exposed to moisture stress conditions. Similar findings were recorded for *Helianthus annus* [33], *Rottboellia cochinchinensis* [34], *Pennisetum glaucum* [35], *Abelmoschus esculentus* [36] and *Amaranthus rudis* [37]. The visible decrease in plant height could be a result of reduced cell enlargement due to low turgor pressure in conditions of drought [1, 11]. *Typha latifolia* and *Stevia rebaudiana*, (Family Asteraceae) also showed reduced shoot height when the seedlings were exposed to drought [38, 39]. Shoot diameter also saw a reduction due to water stress in *Xanthium strumarium* [40] *and Commelina benghalensis* [41].

A root system that increases the capacity of a plant to capture water is a fundamental adaptation to drought [42]. In this study, it was seen that root length increased as drought stress increased. This increase in root length in dry soils and the establishment of a root network that goes deep into the soil would help plants to absorb moisture efficiently, and is one of the mechanisms by which *L*. *serriola* plants tolerate drought stress. The same feature was also observed in *Helianthus annus* and *Catharanthus roseus* [33, 43] where the length of the plant’s root increased when exposed to water stress conditions. A high root to shoot ratio observed in low soil moisture content is another strategic adaptation to develop tolerance to soil moisture deficiency. Thus plants with longer *roots* are able to more effectively compete for soil nutrients and water, while those with a higher proportion of shoots can collect more light energy.

Leaf area plasticity plays a central role in controlling the water use by plants. Hence, a reduction in leaf area due to a plant’s sustained exposure to drought conditions is an important cause of reduced plant yield due to a reduction in the rate of photosynthesis conducted by the plant [44]. Development of optimal leaf area is vital to photosynthesis and in turn dry matter yield. Exposure to water deficit conditions reduced the leaf area in *L*. *serriola* and also in other plant species like *Populus* [45], *Glycine max* [46] and *Anoda cristata* [47].

### Influence of soil moisture on photosynthetic parameters

There was a substantial relation linking treatment with net photosynthesis, stomatal conductance and Fm/Fv value (p< 0.05) with maximum net photosynthesis observed at 100% WHC (Tables 1 and 2). During both the harvests, there was no significant difference in the net photosynthesis of 75% and 100% WHC treatments, however, they were different to the 25% and 50% WHC treatments (p < 0.05) (Tables 1 and 2). Stomatal conductance was found to be significantly different in the 25% and 50% WHC treatments (p < 0.05) (Tables 1 and 2). Fm/Fv value was the highest for the 75% WHC treatment as observed relative to the 100% WHC treatment during both the measurements (Tables 1 and 2). In the first harvest, Fm/Fv value was not significantly different in the 25%, 50% and 100% WHC treatments (p < 0.05) (Table 1). In the second harvest measurement, least Fm/Fv value was observed in the 25% WHC treatment relative to the 100% WHC treatment (Table 2).

When exposed to drought conditions, ion and water transport systems across membranes work to control turgor pressure changes in the guard cells and fuel stomatal closure [5]. This results in lower stomatal conductance and CO_2_ uptake [10]. A decrease in net photosynthesis has also been observed in other weeds, for example *Typha latifolia* [39] and *Parthenium hysterophorus* [28]. An overall reduction of photosynthesis due to lack of adequate water has been linked to both stomatal and non-stomatal limitations [5, 11]. Reduction in stomatal conductance tallied to the reduction in net photosynthesis during soil moisture stress in this study. Thus, it is apparent that stomatal limitation was directly linked to and contributed to the witnessed decrease in photosynthetic capacity, due to decreased CO_2_ availability [48, 49]. Although there was a decrease in net photosynthesis, it was not to a damaging level and the plants coped well enough to develop fully to complete their life cycle under water stress conditions, consistent of the weedy traits of this species.

### Influence of soil moisture on biochemical parameters

**Fig 5:**
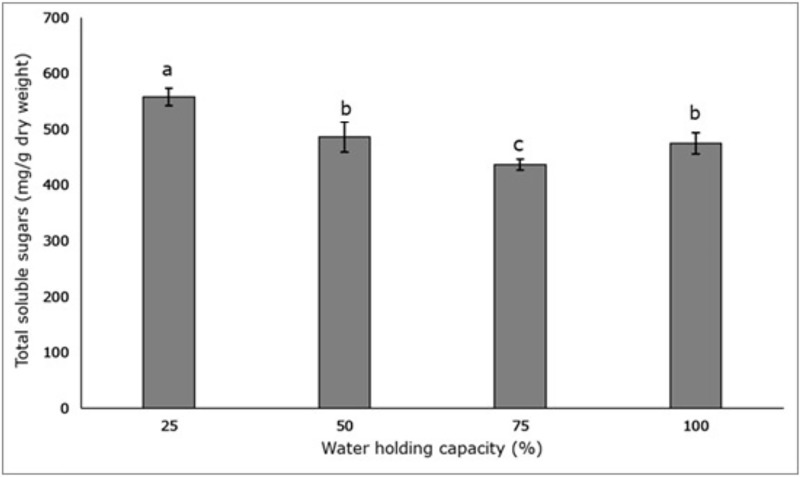
Effect of four different soil moisture levels on the total soluble sugars of *L*. *serriola* measured at first harvest. 25%, 50%, 75% and 100% WHC represent 25%, 50%, 75% and 100% soil water holding capacity in which the plants were maintained. The lines at the top of the bars represent the standard error. Different letters represent significant differences amongst the treatment at first harvest at p < 0.05 (n = 3).

**Fig 6:**
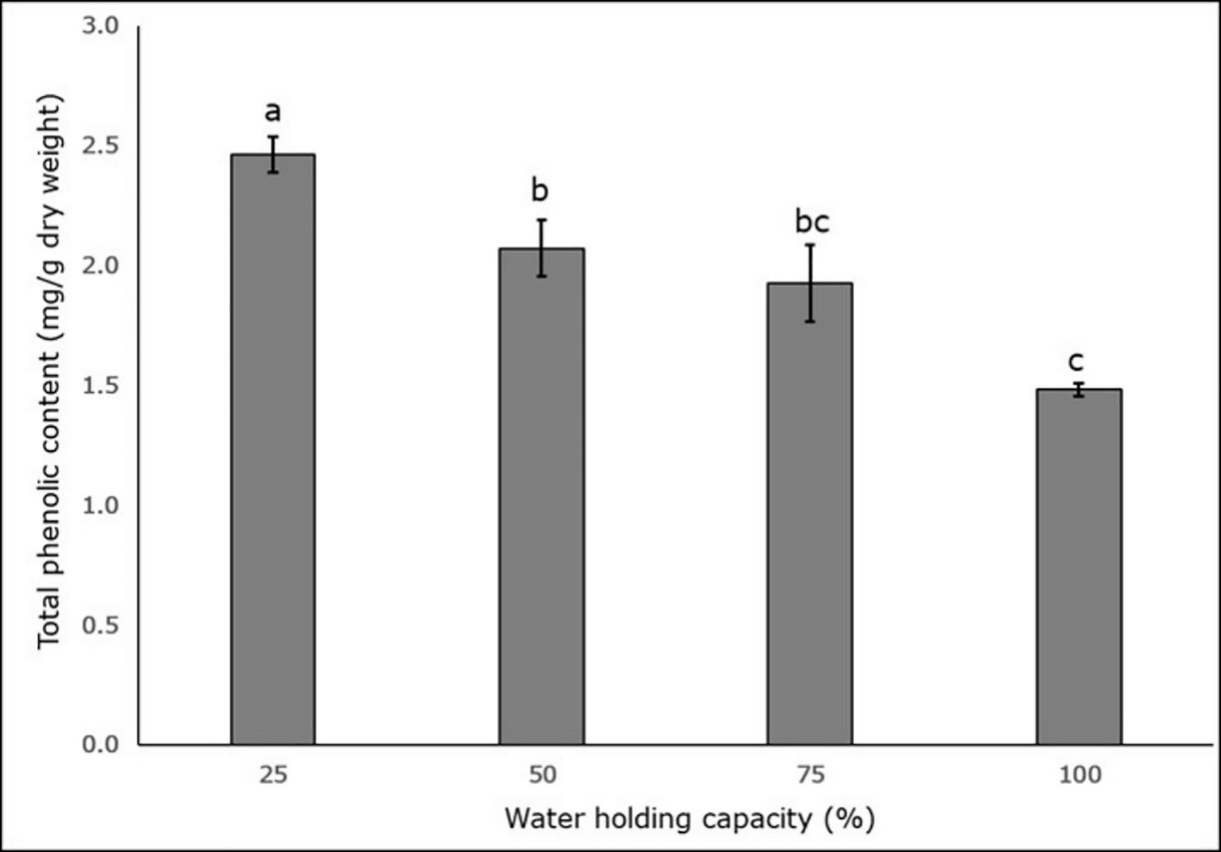
Effect of four different soil moisture levels on the total phenolic content of *L*. *serriola* measured at first harvest. 25%, 50%, 75% and 100% WHC represent 25%, 50%, 75% and 100% soil water holding capacity in which the plants were maintained. The lines at the top of the bars represent the standard error. Different letters represent significant differences amongst the treatment at first harvest at p < 0.05 (n = 3).

Soil moisture stress had a substantial effect on leaf’s biochemistry and total soluble sugars, phenol and chlorophyll content increased as water stress increased (Figs 5 and 6). Total soluble sugars were highest in the 25% WHC treatment (558.24 mg/g dry weight ± 15.88), followed by the 50% (486.70 mg/g dry weight ± 26.57), 100% (475.02 mg/g dry weight ± 19.35) and 75% WHC (437.02 mg/g dry weight ± 10.03) treatments (Fig 5). The 50% and 100% WHC treatments had similar amounts of total soluble sugars, and which were significantly different to the 25% and 75% WHC treatment (p < 0.05) (Fig 5)

The maximum phenolic content was recorded in the 25% WHC treatment (2.46 mg/g dry weight ± 0.07), and lowest phenolic content was in the 100% WHC treatment (1.47 mg/g dry weight ± 0.02) (Fig 6). The total phenolic content in the 25% WHC treatment was 1.6 times higher than that of the 100% WHC treatment. Plants in the 25%, 50% and 100% WHC treatment had significantly different amounts of phenolic compounds (p < 0.05) (Fig 6).

In both harvests, chlorophyll content was maximum in the 25% WHC treatment and minimum in the 75% WHC treatment, relative to the 100% WHC treatment (Tables 1 and 2). During both the harvest, chlorophyll content in the 25% WHC treatment was significantly different to 50% and 100% WHC treatments (p < 0.05) (Tables 1 and 2). Plants in the 75% WHC treatment had the lowest amount of soluble sugars, followed by the 100%, 50% and 25% WHC treatments (Fig 5). This indicates that plants in the 75% WHC treatment were the least stressed, and plants in the 25% WHC treatment were the most stressed. The chemical polysaccharide or starch has an essential role in accumulation of soluble sugars in cells, because its degradation during times of water stress results in an increase of total soluble sugar [50, 51]. The build-up of soluble sugars is well documented and strongly interrelated to the adaptation of becoming drought tolerant in plants [52, 53, 54].

The increase of soluble sugars in the leaves under drought stress in this study would counter the osmotic stress, and is consistent with the findings of other species under drought stress. Examples include *Parthenium hysterophorus* [47], *Zea mays* [51] and *Stevia rebaudiana* [37].

The antioxidant action of phenolic compounds is owing to their redox properties, which play an essential role in neutralizing free radicals, thereby quenching singlet and triplet oxygen or, alternately, decomposing peroxides [55]. Total phenolic content increased in the leaves by 1mg/g, between the 100% and 25% WHC treatments. This would likely provide *L*. *serriola* with a defence mechanism against drought stress (Fig 6). Soluble phenols also increased in *Pisum sativum* [56], *Dolichos lablab* [57], and *Parthenium hysterophorus* [28] in response to drought stress.

### Influence of soil moisture on fecundity

**Fig 7:**
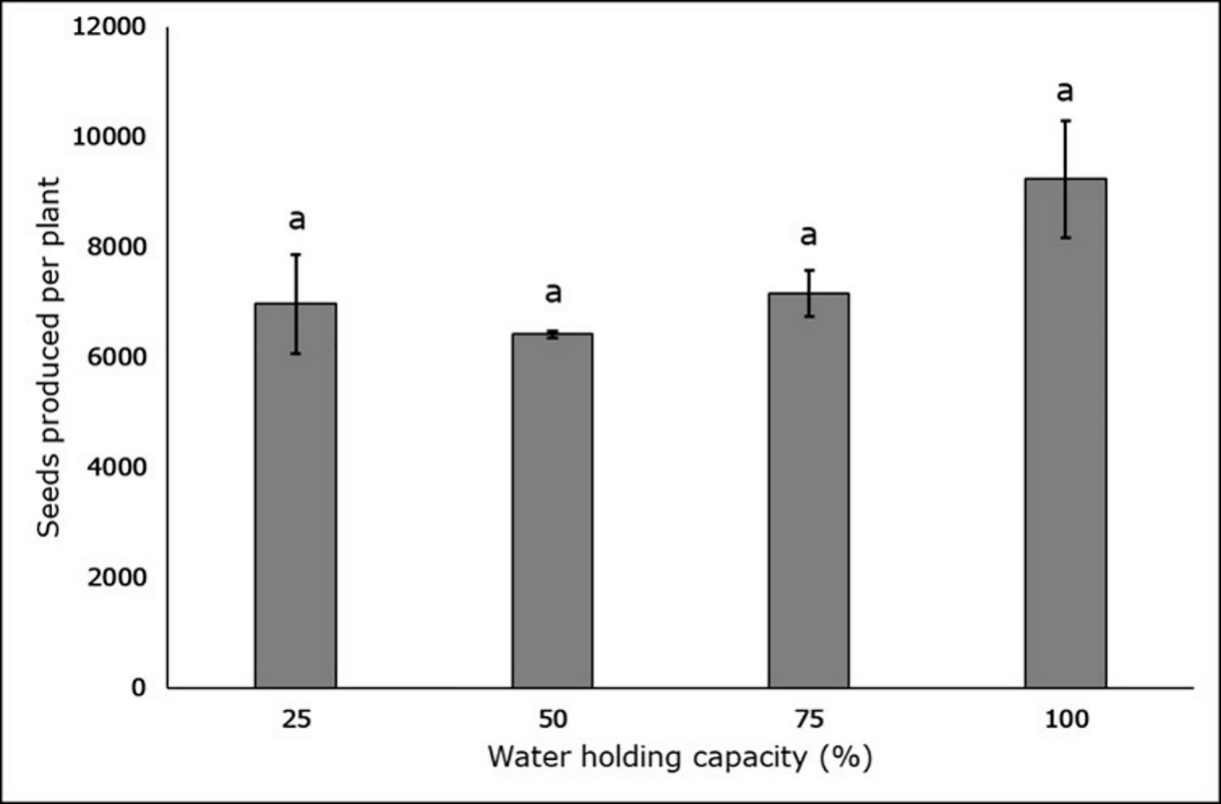
Effect of four different soil moisture levels on the number of seeds produced per plant by *L*. *serriola*. 25%, 50%, 75% and 100% WHC represent 25%, 50%, 75% and 100% soil water holding capacity in which the plants were maintained. The lines at the top of the bars represent the standard error. Different letters represent significant differences amongst the treatment at p < 0.05 (n = 3).

**Fig 8:**
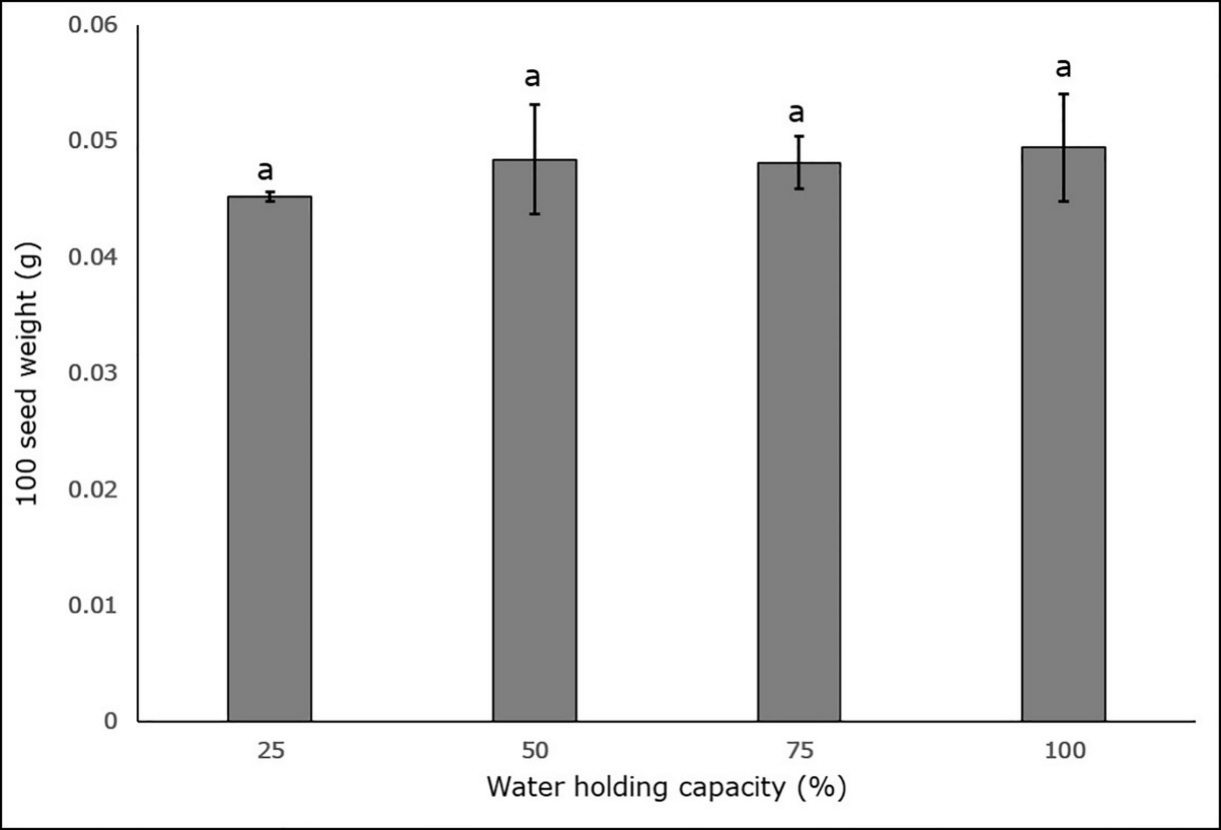
Effect of four different soil moisture levels on 100 seed weight of harvested seeds of *L*. *serriola*. 25%, 50%, 75% and 100% WHC represent 25%, 50%, 75% and 100% soil water holding capacity in which the plants were maintained. The lines at the top of the bars represent the standard error. Different letters represent significant differences amongst the treatment at p < 0.05 (n = 3).

Soil moisture stress had no noteworthy effect on seed production or seed weight in any of the treatments (p < 0.05). Maximum seed production was observed in the 100% WHC treatment (9234.93 ± 1060.08), followed by the 75% (7151.17 ± 419.19), 25% (6968 ± 904.89) and 50% (6415 seeds ± 66.71) WHC treatments (Fig 7). Analysis of the means (p < 0.05) for 100 seed weight indicate no significant variation across all treatments (Fig 8).

**Fig 9:**
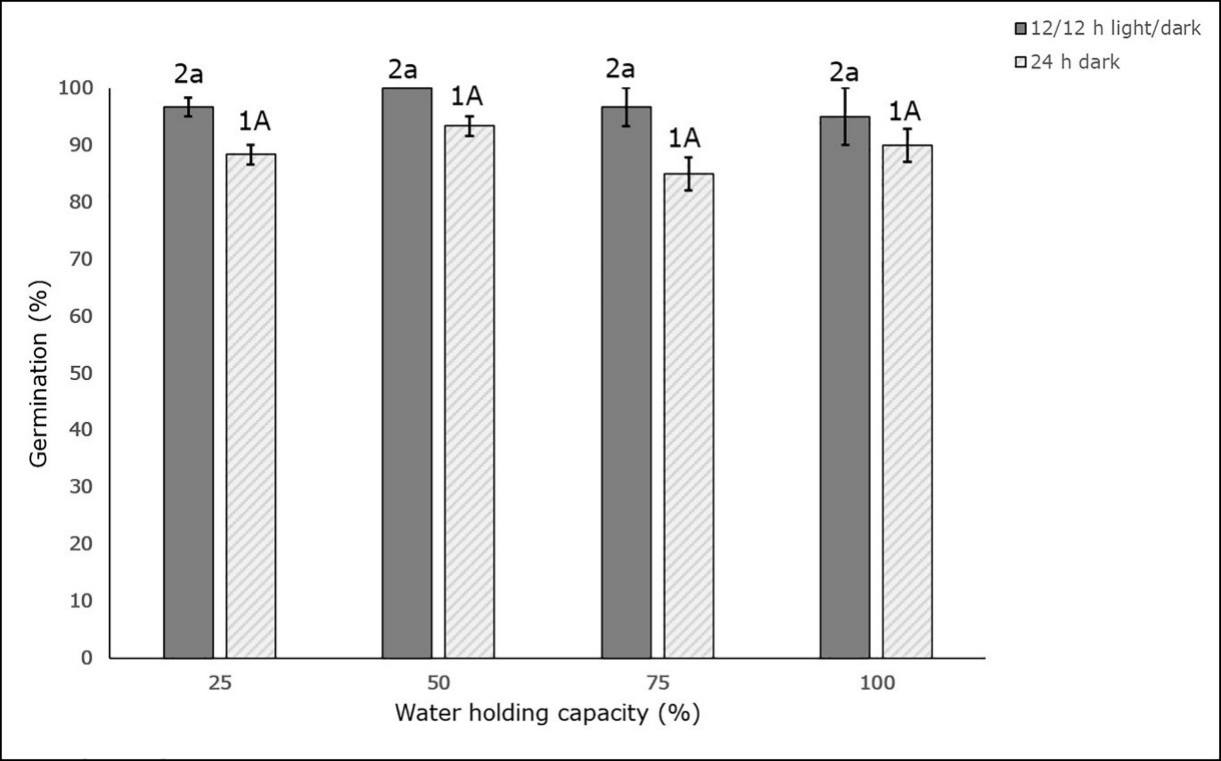
Effect of four different soil moisture levels on germination ability of harvested seeds of *L*. *serriola*. 25%, 50%, 75% and 100% WHC represent 25%, 50%, 75% and 100% soil water holding capacity in which the plants were maintained. The lines at the top of the bars represent the standard error. Different number, small and capital letters represent significant differences amongst the germinating conditions, amongst germination in 12/12 h light/dark condition and amongst germination in 24 h darkness respectively at p < 0.05 (n = 3).

Soil moisture stress did not impact the germination ability of *L*. *serriola* seeds produced under moisture stress. There was no substantial difference (p < 0.05) in the germination percentage of seeds produced by plants exposed to different water holding capacities, when germinated in alternating light (12 hours light/12 hours dark) or continuous darkness (24 hours dark) (Fig 9). However, germination was higher overall in seeds exposed to alternating light, than those germinated in continuous darkness. In the alternating light regime, 100% germination was observed in seeds from plants in the 50% WHC treatment. This was followed by 96.6% for the 75% and 25% WHC treatments, and 95% for the 100% WHC treatment. Germination in the 24 hour dark regime, from highest to lowest, was 93.3%, 90.0%, 88.3%, and 85% for seeds from plants in the 50%, 100%, 25% and 75% WHC treatments, respectively.

Stress factors such as temperature, drought, salinity, and light limitation during a plant’s developmental phase tends to decrease the reproductive allocation to seeds. Whilst this may result in fewer or smaller seeds, it may also influence seed quality traits, such as seed dormancy and chemical defence [19, 20]. Therefore, future shifts in temperature and rainfall may influence seed germination behaviour [58].

In the current study, plants subjected to reduced soil moisture produced up to 25% fewer seeds, compared to plants that were not moisture stressed. However, more than 6000 seeds were produced per plant in all WHC treatments, which is consistent with the weedy traits of this species. This contrasts with observations for *Amaranthus rudis* [37], *Echinochloa colona* [26], *Ambrosia trifida* [27] and *Rottboellia cochinchinensis* [34], where the seed production was reduced with increasing water stress.

Interestingly, no seed dormancy was observed in any of the seeds from plants subjected to any of the water stress treatments, as illustrated in Fig 9. There was also no substantial change or reduction in seed viability according to treatment, since the germination was high to very high, ranging from 85% to 100%. Also, germination of the mother plant seeds has been observed to be greater than 80% at alternating temperatures of 30/20°C (unpublished data). The results obtained from this study are similar to *Echinochloa colona*, where water stress did not reduce the germination of seeds produced from plants subjected to varying levels of water stress [26].

The ability of *L*. *serriola* to develop and propagate and produce seeds in a wide range of soil moisture levels will likely ensure the weed’s endurance in a changeable environment. Seed production is a key factor that impacts weed population dynamics, and it has been noted that the sustainability of any management system will be affected by the amount of seed added to the seed bank over time [59]. Water stress of 25% WHC did not reduce seed production compared to the other soil moisture treatments. This demonstrates that *L*. *serriola* can produce very large amounts of seeds regardless of a deficit of water availability, and therefore will cause a serious infestation in the subsequent growing seasons.

## Conclusion and management recommendations

The above study clearly indicates that *Lactuca serriola* has the adaptability to survive low soil moisture conditions, as low as 25%, while sustaining an important function like seed production close to optimum levels. Exposure of *L*. *serriola* to drought stress resulted in decreased growth rate, and reduced biomass production. A high root to shoot ratio, as well as elevated biomass of the root, allows it to efficiently absorb the required amount of water from the soil and ensure transference to its above-ground parts. An increase in soluble sugars and phenolic content, which are mechanisms to tolerate drought stress was observed as moisture stress increased. Although moisture stress reduced the overall plant biomass, it failed to make any substantial impact on the reproductive abilities of this plant type. While physiological responses were decreased with respect to drought stress, however, the plants were healthy and completed their life cycle even at the low soil moisture level of 25% water holding capacity. Due to its ability to tolerate drought stress, *L*. *serriola* is very likely to expand its range under a drying climate. Its ability to sustain growth through morphological adaptation, physiological and biochemical regulation, even during times of water stress, ensures *L*. *serriola* with a robust mechanism to continue spreading to new regions.

It is recommended that management strategies for *L*. *serriola* should include early control of this weed in cropping fields, because the combined pressure of enhanced weed competition and water stress conditions could severely impair crop yield. Thus it is important to control *L*. *serriola* at the earliest possible time in order to conserve soil moisture for crops where possible.

## Supporting information

S1 File Raw data inputs for this study. Data for repeated measures, biochemical analysis, fecundity, 1^st^ harvest and 2^nd^ harvest are provided in the excel sheet in their respective tabs.
(XLSX)
